# Lipoproteins predicting coronary lesion complexity in premature coronary artery disease: a supervised machine learning approach

**DOI:** 10.3389/fcvm.2025.1470500

**Published:** 2025-04-24

**Authors:** Marta Marcinkowska, Agnieszka Kuchta, Petra Małgorzata Grešner, Tomasz Figatowski, Piotr Kasprzyk, Radosław Targoński, Wojciech Sobiczewski, Miłosz Jaguszewski, Marcin Fijałkowski, Marcin Gruchała, Agnieszka Mickiewicz

**Affiliations:** ^1^ First Department of Cardiology, Medical University of Gdansk, Gdansk, Poland; ^2^ Department of Clinical Chemistry, Medical University of Gdansk, Gdansk, Poland; ^3^ Centre of Biostatistics and Bioinformatics Analyses, Medical University of Gdansk, Gdansk, Poland

**Keywords:** premature coronary artery disease, lipoprotein(a), SYNTAX score, machine learning, LDL - cholesterol

## Abstract

**Introduction:**

We aimed to assess the usefulness of lipoprotein(a) [Lp(a)] and LDL-C levels as potential predictors of coronary lesions' complexity in patients with premature coronary artery disease (pCAD).

**Methods:**

This study enrolled 162 consecutive patients with pCAD undergoing coronary angiography. The SYNTAX score (SS) was used to assess coronary lesions' complexity. Linear discriminant analysis (LDA) was employed to construct a multivariate classification model enabling the prediction of coronary lesions' complexity in SS.

**Results:**

The Lp(a) levels among patients with SS ≥ 23 and with SS 1-22 were significantly higher than those with SS = 0 (*p* = 0.021 and *p* = 0.027, respectively). The cut-off point for the Lp(a) level of 63.5 mg/dl discriminated subjects with SS ≥ 23 from those with SS ≤ 22 (sensitivity 0.546, specificity 0.780; AUC 0.620; *p* = 0.027). An LDA-based model involving the Lp(a) level, age, sex and LDL-C provided improved discrimination performance (sensitivity 0.727, specificity 0.733, AUC 0.800; *p* = 0.0001).

**Conclusions:**

Lp(a) levels in pCAD patients are associated with the advancement of coronary artery lesions in SS patients. An Lp(a) level of 63.5 mg/dl can be the cut-off point for the identification of subjects with SS ≥ 23. LDA-based modelling using Lp(a), LDL-C, age and gender may be an applicable tool for the preliminary identification of patients at risk of more complex coronary artery lesions.

## 1 Introduction

Premature coronary artery disease (pCAD) leads to transient or permanent disability and mortality in working-age patients, challenging the public health system. Despite significant diagnostic and therapeutic advances, published data show an increase in the number of young patients hospitalized for premature myocardial infarction. Moreover, trends indicating reduced mortality in coronary artery disease are less pronounced in patients aged <55 years, compared to older groups (1, 2). Therefore, the early identification of all modifiable cardiovascular (CV) risk factors and prompt therapeutic interventions are of particular importance.

Lipoprotein(a) [[Lp(a)] with proatherogenic, proinflammatory, prothrombotic and antifibrinolytic effects is considered an independent risk factor for the development and progression of atherosclerotic cardiovascular disease (ASCVD) (3–8). Lp(a) has an almost 2–3 times greater ability to accumulate in areas of endothelial damage than LDL particles (9). Epidemiological and Mendelian randomization studies illustrated a causal relationship between Lp(a) and CAD, peripheral artery disease (PAD), stroke, the progression of aortic stenosis, heart failure and CV mortality, regardless of low-density lipoprotein cholesterol (LDL-C), non-high-density lipoprotein cholesterol (non-HDL-C) and other CV risk factors (4, 10–18). Moreover, Lp(a) is responsible for a residual CV risk, even among optimally treated patients with an LDL-C less than 70 mg/dl (19).

It is estimated that up to 90% of Lp(a) plasma concentrations are genetically determined, leading to premature ASCVD and an increased family prevalence of premature cardiovascular disease. According to genome-wide association studies, the main determinant of Lp(a) serum levels is LPA (6q26-27), a gene encoding apo(a) characterized by significant size polymorphism and the presence of single nucleotide polymorphisms (SNPs) (20–22). Current guidelines do not recommend routine genetic testing in patients with hyper-Lp(a) in both the primary and secondary prevention of CAD (23).

Published data have reported that the Lp(a) levels of patients with early manifestations of ASCVD are higher compared to those diagnosed at a typical age, determining the more rapid progression of pCAD independent of a positive family history of ASCVD (24–26). Analyses of UK Biobank patients aged 40–69 years (57.0 ± 8.1 years) observed by 11-year period showed that higher levels of Lp(a) predicted the risk of an ASCVD event in both primary and secondary prevention. An increase in Lp(a) of 50 nmol/L (approximately 23 mg/dl) was associated with a hazard ratio for an ASCVD event of 1.11 (95% CI, 1.10–1.12) (27). In patients aged ≤60 years, elevated Lp(a) is an independent risk factor for acute coronary syndrome (ACS). In young patients aged ≤45 with a concentration of >50 mg/dl, the risk of ACS increases as much as 3 times (28).

According to guidelines, CAD is a pathological process characterized by atherosclerotic plaque accumulation in the epicardial arteries, whether obstructive or non-obstructive (29). Lp(a) is also strongly correlated with the severity and complexity of coronary stenoses, measured in a coronary angiography with the SYNTAX Score (Synergy between Percutaneous Coronary Intervention with Taxus and Cardiac Surgery Score; SS), even in the presence of normal total cholesterol (TC) levels (30–32). Chen et al. demonstrated that Lp(a) predicted the severity of new-onset CAD independent of other ASCVD risk factors (33).

SS is a comprehensive, multi-stage angiographic scoring system designed to assess the number, advancement, location, complexity and functional significance of atherosclerotic lesions. According to ESC/EACTS guidelines for myocardial revascularization, calculating the SS includes 11 steps. The SS point value determines the coronary revascularization strategy. In patients in a stable clinical condition with advanced coronary lesions reflected in a high SS score, a percutaneous coronary intervention (PCI) should not be performed *ad hoc*, and invasive treatments should be discussed by the cardiogroup. A multivessel coronary artery disease (MVD) and an SS value of 0–22 points support a percutaneous coronary intervention strategy, while an MVD with an SS ≥ 23 points suggests the anatomy of coronary artery lesions, likely resulting in an incomplete revascularization with PCI. The SS ≥ 23 points should favor a coronary artery bypass graft (CABG) strategy. Moreover, in the case of high-scoring left main stem disease (LMSD) – SS ≥ 33 points, the PCI strategy is contraindicated (III B) (32).

Additionally, SS is an independent predictor of major adverse cardiac and cerebrovascular events, as well as long-term mortality, in patients with LMSD or MVD undergoing PCI (34–38).

The validity of using machine learning methods in the context of searching for non-invasive, economical, fast and adequate techniques for early CAD diagnostics, being an alternative to classical coronary angiography, has been emphasized in previous studies (39). Sayadi M. et al. underlined the significance of appropriate feature selection, presenting a substantial impact on the machine-learning performance for detection of CAD using noninvasive clinical parameters (40). Koloi A. et al. posited that predictions of early stage angiographic CAD could be obtained using a set of routine laboratory markers, age, gender, and smoking status (41). There are several predictive models, which can be applied for classification in clinical studies: logistic regression, decision trees, neural networks and linear discrimination (LDA). Among them LDA can serve as a suitable classification tool. This well-established, interpretable method is widely used in life sciences research for classifying subjects into groups based on multiple predictors. LDA offers relatively straightforward interpretation of how predictor variables contribute to classification, which we believe is especially important for clinical audiences.

We aimed to evaluate the association between Lp(a), LDL-C levels and coronary atherosclerosis stages measured by SS in pCAD patients with a supervised machine learning approach and to determine their potential roles in predicting the angiographic severity of CAD.

## 2 Materials and methods

### 2.1 Study design

This study enrolled 162 consecutive patients hospitalized with a pCAD diagnosis at the First Cardiology Department of the University Clinical Centre in Gdansk between 2019 and 2022.

In patients without a history of ASCVD, the diagnosis of CAD was based on the visualization of atherosclerotic plaque in the coronary angiography performed during hospitalization. In patients with a history of CAD, the diagnosis was based on available medical records, considering an episode of previous ACS, previous coronary revascularization (PCI, CABG) or the presence of atherosclerotic plaque detected in imaging tests (previous coronary angiography or computed tomography angiography). The diagnosis of pCAD was made in men under the age of 55 years and in women under age 65 (29, 42).

This study was approved by the Independent Ethics Committee for Research Studies at the Medical University of Gdansk (NKBBN/50/2019). Before enrolment in the study, all patients provided written consent to participate in the project in accordance with the approved protocol.

This research excluded patients with known heart failure and a reduced left ventricle ejection fraction (LVEF) below 30%, chronic kidney disease (CKD) stages G4–G5 and/or high inflammatory parameters defined as C-reactive protein (CRP) levels above 30 mg/L, and hypertriglyceridemia with triglyceride (TG) levels >400 mg/dl (43).

### 2.2 Clinical data

Clinical data were collected, including the following: reason for admission, detailed history of cardiovascular disease (CVD) and other comorbidities, CV risk factors and previous results of diagnostic tests and treatment, including pharmacological treatment, with particular emphasis on lipid-lowering therapy (LLT). In patients without a history of CAD, the age of diagnosis was determined by the presence of atherosclerotic lesions in a coronary angiography performed during hospitalization. In the case of a CAD history, the age of diagnosis was established based on available medical records, considering past ACS episodes, previous coronary revascularization [PCI, CABG] or the presence of plaque found on imaging examinations (prior coronary angiography or coronary computed tomography angiography). A positive family history of premature CVD was defined as a fatal or non-fatal CV event and/or CVD diagnosed in a first-degree relative aged <55 years for males or <65 years for females (29). Recurrent cardiovascular events (RCVEs) were defined as a history of a minimum of 2 ACS episodes and/or evidence of concomitant atherosclerosis in a non-coronary arterial vascular bed (cephalic or lower extremity arteries). Hypercholesterolaemia was diagnosed based on the history of the use of LLT or an LDL-C level ≥55 mg/dl (44). Atherosclerosis in another arterial bed was defined as a symptomatic stenosis of more than 50% of the luminal cross-section or an asymptomatic stenoses greater than 70%, found on imaging or in a previous vascular procedure (45–47). The diagnosis of other comorbidities, including hypertension (HT) and diabetes mellitus (DM), was confirmed based on the available medical records and according to accepted standards (48). The definition of overweight and obesity was based on body mass index (BMI), as recommended by the WHO (49). A history of smoking was defined as active smoking upon entry to the study or smoking history for at least one year. The classification of CKD was based on an assessment of the glomerular filtration rate (GFR).

All patients underwent coronary angiography using the Judkins technique, as clinically indicated. The number and severity of coronary lesions and their location, complexity and functional significance were assessed based on SS using an online calculator (http://syntaxscore.org) in 149 patients. The low technical quality of coronary angiography recordings in 13 patients prevented SS assessments. For patients with a history of PCI or CABG, the analysis was based on the earliest available angiography. In 53 patients, the SS results were determined based on a coronary angiography before admission. Two experienced operators independently evaluated the SS calculations and angiographic results. In the case of inconsistency, a supervising cardiologist performed a third assessment. Moreover, SS-related indices were estimated: residual SS and SYNTAX Revascularization Index. Based on the SS score, patients were included in one of 3 groups: SS = 0 points (*n* = 42), SS 1–22 points (*n* = 84) or SS ≥ 23 points (*n* = 23), then to one of 2 groups: SS ≤ 22 points (*n* = 126) or SS ≥ 23 points (*n* = 23).

The biological material for biochemical tests was obtained by collecting peripheral venous blood in a fasting state (after at least 12 hours of fasting) during routine diagnostic procedures. The biochemical tests were performed at the Central Clinical Laboratory, University Clinical Centre in Gdansk. All samples were analyzed in a single laboratory and Lp(a) was measured directly. The Lp(a) concentration was determined using a Siemens system employing the latex immunonephelometric method (Siemens N Latex). The lipid profile was obtained based on enzymatic tests. LDL-C was calculated according to the Friedewald formula (43). Other routine biochemical assays were garnered using standard validated diagnostic methods for individual parameters.

Patients underwent long-term clinical follow-up and the occurrence of major adverse cardiovascular events (MACE) was assessed. MACE was defined as: all-cause death, cardiac death, myocardial infarction and repeated revascularization.

### 2.3 Statistical analysis

All statistical calculations were performed using R software (50). The distribution of quantitative data was verified with the Shapiro–Wilk W test. Depending on the normality of distribution, quantitative data were then presented as mean ± standard deviation or median with interquartile range (IQR). Qualitative data were presented as numbers and percentages. The significance of inter-group differences was tested using the Mann–Whitney *U* test or Kruskal–Wallis H test, depending on the number of compared groups. For categorical variables, the significance of inter-group differences was tested using Pearson's chi-square test or Fisher's exact test, depending on the resultant group sizes. Simple correlations were assessed using Spearman's rank correlation coefficient (R_SP_).

The association between qualitative dependent variables (“SYNTAX Score classification”) and plausible predictors was modeled using the Linear Discriminant Analysis (LDA) method with an exhaustive stepwise procedure examining all possible models of the second to the eleventh order of dimensionality (the highest order was dictated by the number of potential predictors and/or confounders) (51, 52). All the examined models were trained on a training set representing 70% of the overall dataset and then characterized by the classification error obtained from the remaining 30% of the set (the test set). To increase the robustness of the obtained models to non-random split bias, this procedure was repeated 50 times. No validation set was used due to limited study size and the simplicity of used model. Ultimately, each model was characterized by a mean classification error on the test set (Table 1). The model with the lowest mean classification error was then selected as the best classification model and thoroughly examined by receiver operating characteristic (ROC) analysis to determine the optimal threshold value providing the best classification performance. The obtained ROC curve was also used to derive the most important measures of predictive and classification performance of the model, including the area under the curve (AUC), sensitivity and specificity. Two-sided tests were used in all analyses, and the statistical significance was assumed to be *p* < 0.05.

**Table 1:** Basic statistical characteristics of model 2.

Order	Mean LDF1 values in the SS ≤ 22 group	Mean LDF1 values in the SS ≥ 23 group	*p*	Classification error in the training set	Classification error in the test set
4	−0.181	0.986	0.00043	0.13677	0.15953

Abbreviations: SS, SYNTAX score.

LDA analysis was based on the *MASS* and *candisc* packages using their basic *lda()* and *candisc()* functions, without modifications. The analysis of ROC curves was based on the *pROC* package using its basic *roc()* function.

## 3 Results

### 3.1 Clinical and biochemical characteristics

The study enrolled 86 men aged 55.7 years (IQR 49–62) and 76 women aged 60.3 years (IQR 58–64); *p* < 0.001; hospitalized for chronic coronary syndromes (60%) or ACS. *De novo* CAD was diagnosed during hospitalization in 38% of the patients. The age at which CAD was diagnosed was 46.8 (IQR 43–52) and 56.6 (IQR 54–62) years in men and women, respectively; *p* < 0.001. Each subject had at least 2 CV risk factors. The clinical and biochemical characteristics of the study group are presented in Supplementary Tables S1, S2.

The concentration of Lp(a) in the study population was 25 mg/dl (IQR 8–69.5), with statistically insignificant differences between males and females (*p* = 0.324) and between various reasons for hospitalization (*p* = 0.382). No statistically significant correlation was found between the Lp(a) concentration and the age of pCAD diagnosis (R_S*P*_ = −0.026; *p* = 0.74) and the premature clinical manifestation of atherosclerosis in other (non-coronary) arterial beds (R_S*P*_ = −0.037; *p* = 0.64). However, the difference in Lp(a) concentrations between the RCVE and non-RCVE groups [29 (IQR 11–85) mg/dl vs. 22 (IQR 8–56) mg/dl] was marginally significant (*p* = 0.063).

**Figure 1:**
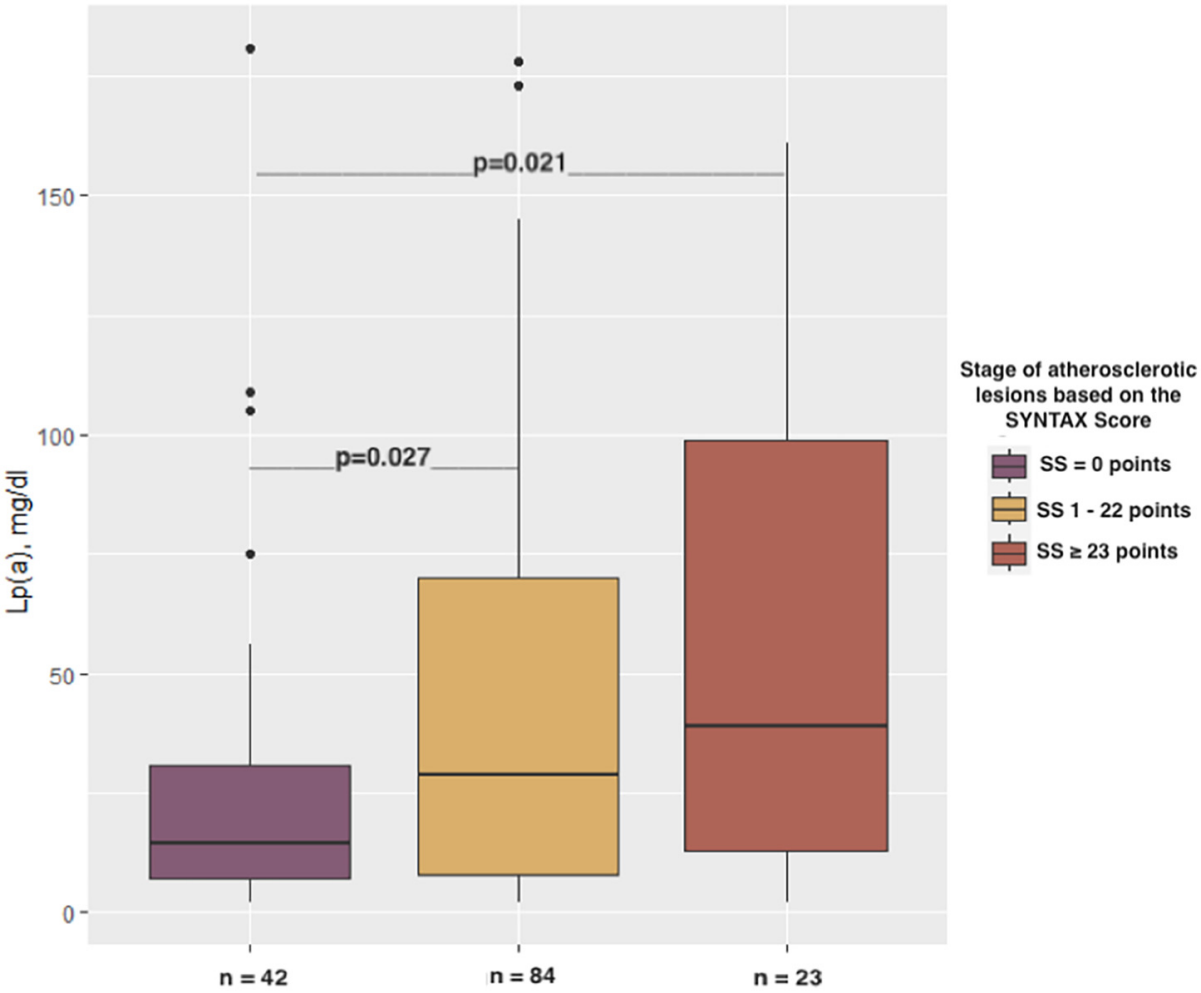
Relationship between the stages of atherosclerotic lesions based on the SYNTAX score and Lp(a) concentration.

Lp(a) levels varied significantly depending on the complexity of epicardial lesions, as determined by SS (*p* = 0.026). The Lp(a) concentrations among patients with SS ≥ 23 points [39 (IQR 13–99) mg/dl] as well as patients with 1–22 SS points [29 (IQR 8–70.2) mg/dl] were found to be notably higher compared to those found among patients with 0 SS points [14.5 (IQR 7.2–31) mg/dl; *p* = 0.021 and *p* = 0.027, respectively; Figure 1].

The SS = 0 points revealed a markedly higher frequency of coronary revascularization (71.4% vs. 47.6%; *p* < 0.05) as well as a positive family history of pCAD (52.4% vs. 28.6%; *p* < 0.05) compared to the SS 1–22-point group. No statistically significant differences in the incidence of other major cardiovascular risk factors (HT, DM, overweight/obesity, smoking) as well as age (*p* = 0.737) and gender (*p* = 0.324) were found between the identified groups (SS = 0 points, SS 1–22 points, SS ≥ 23 points).

There were no notable differences in lipid profile (TC, LDL-C, HDL-C, TG), renal function (creatinine, GFR), liver function [alanine transaminase (ALT)], inflammation (CRP), uric acid and glucose levels in either group; *p* > 0.05; Table 2.

**Table 2:** Biochemical characteristics of patients with pCAD according to the severity of their coronary atherosclerotic lesions based on SS (*n* = 149).

Parameter	SYNTAX score			*p*
	SS = 0 pts *n* = 42 [median, IQR]	SS 1–22 pts *n* = 84 [median IQR]	SS ≥ 23 pts *n* = 23 [median IQR]	
TC [mg/dl]	161.0 (136.0–198.0)	166.5 (137.0–194.5)	175.0 (145.0–241.5)	0.5030
LDL-C [mg/dl]	80.0 (69.0–112.00)	92.0 (71.0–124.5)	100.0 (79.0–151.0)	0.2520
HDL-C [mg/dl]	47.0 (38.0–61.0)	46.0 (38.6–53.0)	38.0 (33.5–54.0)	0.1540
non-HDL-C [mg/dl]	102.0 (91.0–152.0)	118.5 (95.5–145.5)	127.00 (105.5–184.0)	0.2000
TG [mg/dl]	116.0 (83.0–165.0)	113.0 (83.0–153.5)	144.0 (91.5–194.5)	0.2120
Creatinine [mg/dl]	0.8 (0.7–1.0)	0.8 (0.7–1.0)	0.8 (0.7–1.0)	0.9960
GFR [ml/min/1.73 m^2^]	87.5 (74.5–90.0)	90.0 (83.0–90.0)	90.0 (75.0–90.0)	0.1440
ALT [IU/L]	26.0 (21.0–31.5)	27.0 (20.0–34.3)	27.0 (17.0–33.0)	0.8770
CRP [mg/L]	1.75 (0.9–3.2)	1.6 (0.8–2.9)	2.6 (0.8–7.4)	0.2940
Uric acid [mg/dl]	5.8 (4.6–7.5)	5.6 (5.1–6.5)	5.4 (4.7–6.1)	0.5150
Glucose [mg/dl]	100.0 (93.0–128.0)	99.0 (91.0–114.0)	103.00 (94.3–123.3)	0.6590

Abbreviations: ALT, alanine transaminase; CRP, C-reactive protein; GFR, glomerular filtration rate; HDL-C, high-density lipoprotein-cholesterol; IQR, interquartile range; LDL-C, low-density lipoprotein-cholesterol; non-HDL-C, non-high-density lipoprotein-cholesterol; SS, SYNTAX score; TC, total cholesterol; TG, triglycerides.

Moreover, the achievement of the therapeutic goal, i.e., the reduction of the LDL-C concentration below 55 mg/dl, was not demonstrated to be associated with the severity of atherosclerotic lesions in coronary angiography; *p* = 0.756. The distinction above was also not observed in patients treated and untreated with statins before hospitalization; *p* = 0.519. Tables 2, 3 present the detailed clinical and biochemical characteristics of the pCAD patients.

**Table 3:** Clinical characteristics of patients with pCAD according to the severity of their coronary atherosclerotic lesions based on SS (*n* = 149).

Parameter	SYNTAX score			*p*
	SS = 0 pts *n* = 42	SS 1–22 pts *n* = 84	SS ≥ 23 pts *n* = 23	
	*n* [%]	*n* [%]	*n* [%]	
Hypertension	33 [78.6]	58 [69.1]	21 [91.3]	0.0800
Overweight (BMI ≥ 25 < 30)	16 [38.1]	32 [38.1]	12 [52.2]	0.4487
Obesity (BMI ≥ 30)	23 [54.8]	30 [35.7]	10 [43.5]	0.1238
Male gender	24 [57.1]	46 [54.8]	15 [65.2]	0.324
Smoking	23 [54.8]	32 [38.1]	12 [52.1]	0.2469
DM2	15 [35.7]	18 [21.4]	10 [43.5]	0.0604
DM1	0 [0.0]	1 [1.2]	1 [4.4]	0.3639
Positive family history of pCAD	22 [52.4]	24 [28.6]	8 [34.8]	0.0253
Prior ACS	22 [52.4]	33 [39.3]	12 [52.2]	0.2849
Prior coronary revascularization	30 [71.4]	40 [47.6]	15 [65.2]	0.0271
Other ASCVD	6 [14.3]	12 [14.3]	5 [21.7]	0.6611
Prior stroke/TIA	1 [2.4]	4 [4.8]	1 [4.4]	0.8625
Carotid atherosclerosis	1 [2.4]	3 [3.6]	1 [4.4]	0.6691
PAD	4 [9.5]	6 [7.1]	3 [13.0]	0.5618
CKD stage G3	3 [7.1]	7 [8.3]	4 [17.4]	0.3563
Asthma	2 [4.8]	7 [8.3]	1 [4.4]	0.9017
COPD	1 [2.4]	4 [4.8]	1 [4.4]	0.8625
Previous LLT	36 [85.7]	60 [71.4]	18 [78.3]	0.1993
LDL-C < 55 mg/dl	4 [9.5]	7 [8.3]	2 [8.7]	0.9229
LDL-C ≥ 55 mg/dl	37 [88.1]	77 [91.7]	21 [91.3]	0.9229
Hypercholesterolaemia	42 [100.0]	84 [100.0]	23 [100.0]	<0.0001

Abbreviations: ACS, acute coronary syndrome; ASCVD, atherosclerotic cardiovascular disease; BMI, body mass index; CKD, chronic kidney disease; COPD, chronic obstructive pulmonary disease; DM1, type 1 diabetes mellitus; DM2, type 2 diabetes mellitus; LDL-C, low-density lipoprotein-cholesterol; LLT, lipid-lowering therapy; PAD, peripheral arterial disease; pCAD, premature coronary artery disease; PCI, percutaneous coronary intervention; SS, SYNTAX score; TIA, transient ischemic attack.

**Figure 2:**
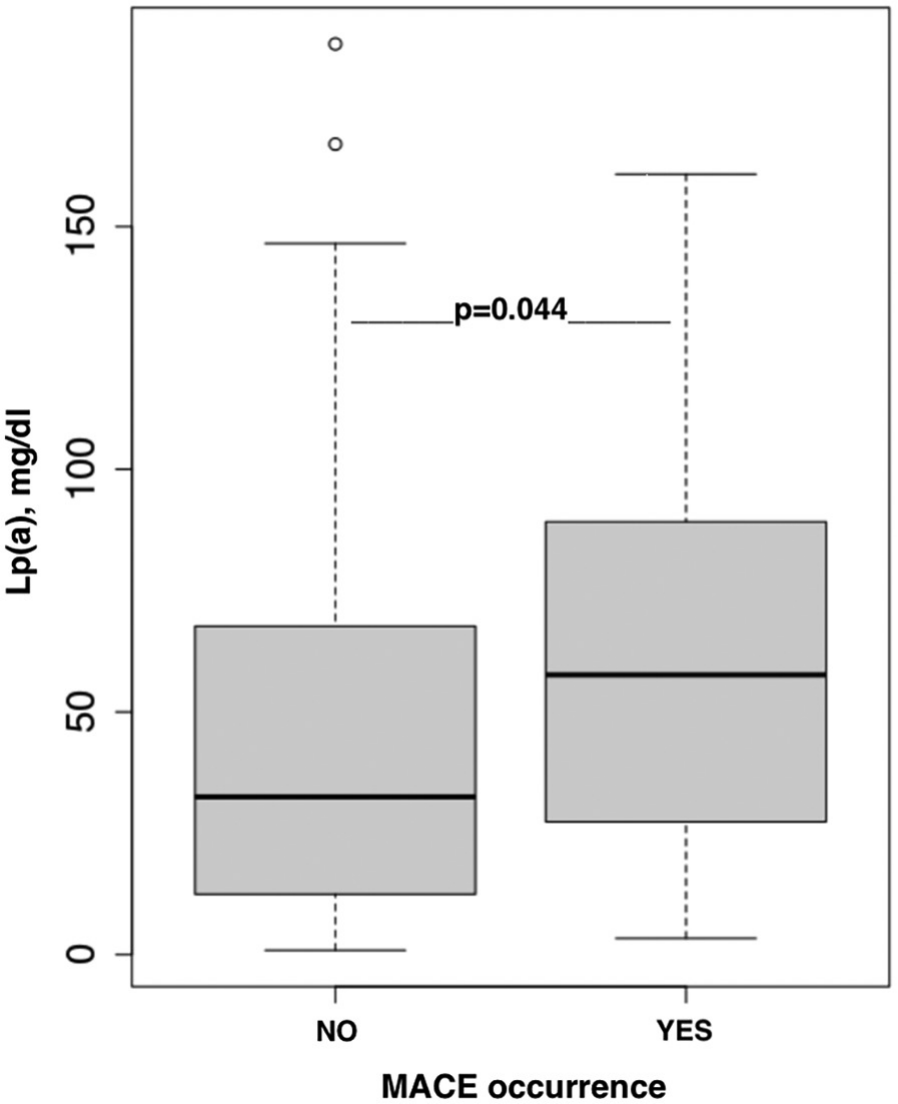
The association between Lp(a) concentration and MACE occurrence.

A significant association was found between Lp(a) levels above the median and the occurrence of MACE [HR 2.1 (1–4.4); *p* = 0.044; Figure 2] in the follow-up of 5 (IQR 3–7.25) months.

### 3.2 Simple univariate model—model 1

**Figure 3:**
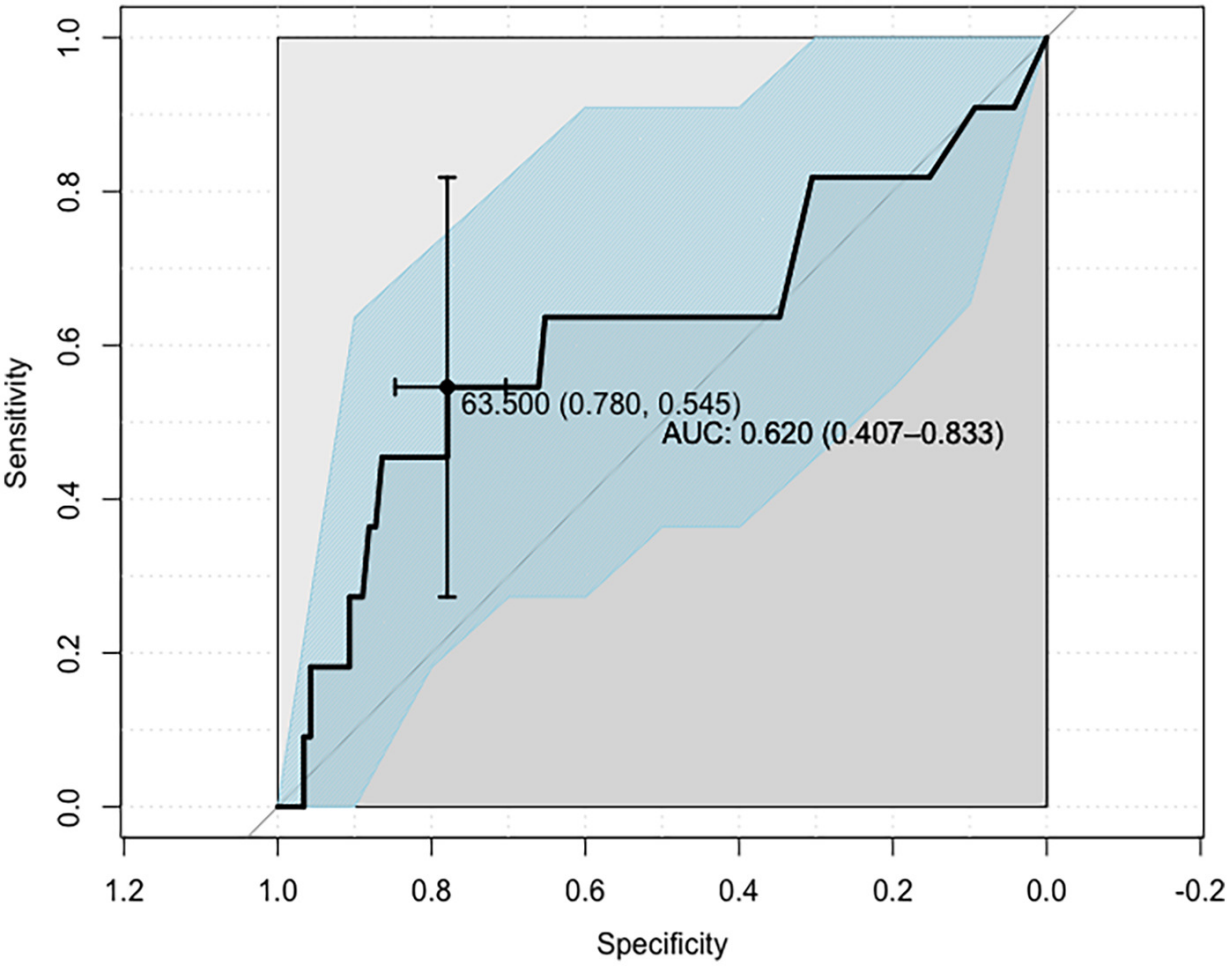
ROC curve defining the Lp(a) concentration cut-off point for the indicated SYNTAX score groups, identifying patients with advanced CAD.

Subsequently, ROC analysis was employed to determine the Lp(a) concentration cut-off value enabling the best possible discrimination of the SS ≥ 23 subjects from those of the SS = 0 and SS ∈{1;22} groups (hereby denoted SS ≤ 22; *n* = 123). The Lp(a) cut-off value was determined to be 63.5 mg/dl and was found to provide the proper classification of SS ≥ 23 subjects with only an overall moderate performance (AUC: 0.620, 95% CI: 0.407–0.833; sensitivity: 0.546, 95% CI: 0.273–0.818; specificity: 0.780, 95% CI: 0.703–0.848). Nevertheless, such a classifier can still be assumed to improve the assigning of patients to one of the abovementioned two groups (OR = 4.2, 95% CI: 1.2–15.0; *p* = 0.027; Figure 3). Individuals with Lp(a) >63.5 mg/dl are classified into the SS ≥ 23 category, whereas subjects with Lp(a) <63.5 mg/dl into the SS ≤ 22 category.

### 3.3 Multivariate modelling—model 2

To seek the best Lp(a)-based covariate-enhanced multivariate model allowing better SS ≥ 23 subject discrimination from others, the Lp(a) concentration and 10 additional confounders [including age, sex, BMI, smoking, LDL-C level, statin use, HT, DM (Type 1 or 2), positive family history of CAD and stage G3 CKD] were further subject to the LDA modelling procedure. Here, an exhaustive examination of all possible models from the second to eleventh order of dimensionality was performed. In total, we examined 2,038 LDA models, out of which the lowest mean classification error (16.0%) on a test set was found for a fourth-order model consisting of Lp(a) concentration, as well as sex, age and LDL-C concentration as covariates:

LDF1=0.0095×Lp(a)+0.0931×age−1.2149×sex(female=1/male=0)+0.0125×LDL−C

The LDF1 value was directly proportional to the concentration of Lp(a) and LDL-C. The age of the individual has a roughly 10-fold greater contribution to this value (also a directly proportional relationship). However, the sex of the subject had the strongest influence on the LDF1 value: in women, the LDF1 value was significantly lowered, which results in a substantial decrease of the likelihood of classifying females into SS ≥ 23 group.

Model 2 reached a high level of statistical significance (*p* = 0.0001), and subsequent ROC analysis identified the −0.363 value as the LDF1 (linear discriminant function) cut-off value, ensuring evenly improved overall performance compared to the above-described simple univariate Lp(a)-based model—Model 1 (AUC: 0.797, 95% CI: 0.701–0.894; sensitivity: 0.727, 95% CI: 0.546–0.909; specificity: 0.733, 95% CI: 0.650–0.808).

Evenly improved overall performance was demonstrable mainly in the AUC value, significantly changed from 0.62 (model 1) to 0.80 (model 2). The sensitivity of model 2 increased from 0.55 to 0.73, the specificity value slightly decreased from 0.78 to 0.73. Model 2 provides a balanced ratio of sensitivity and specificity consequently. The PPV of model 2 increased from 0.19 to 0.33 and the NPV decreased from 0.95 to 0.94.

**Figure 4:**
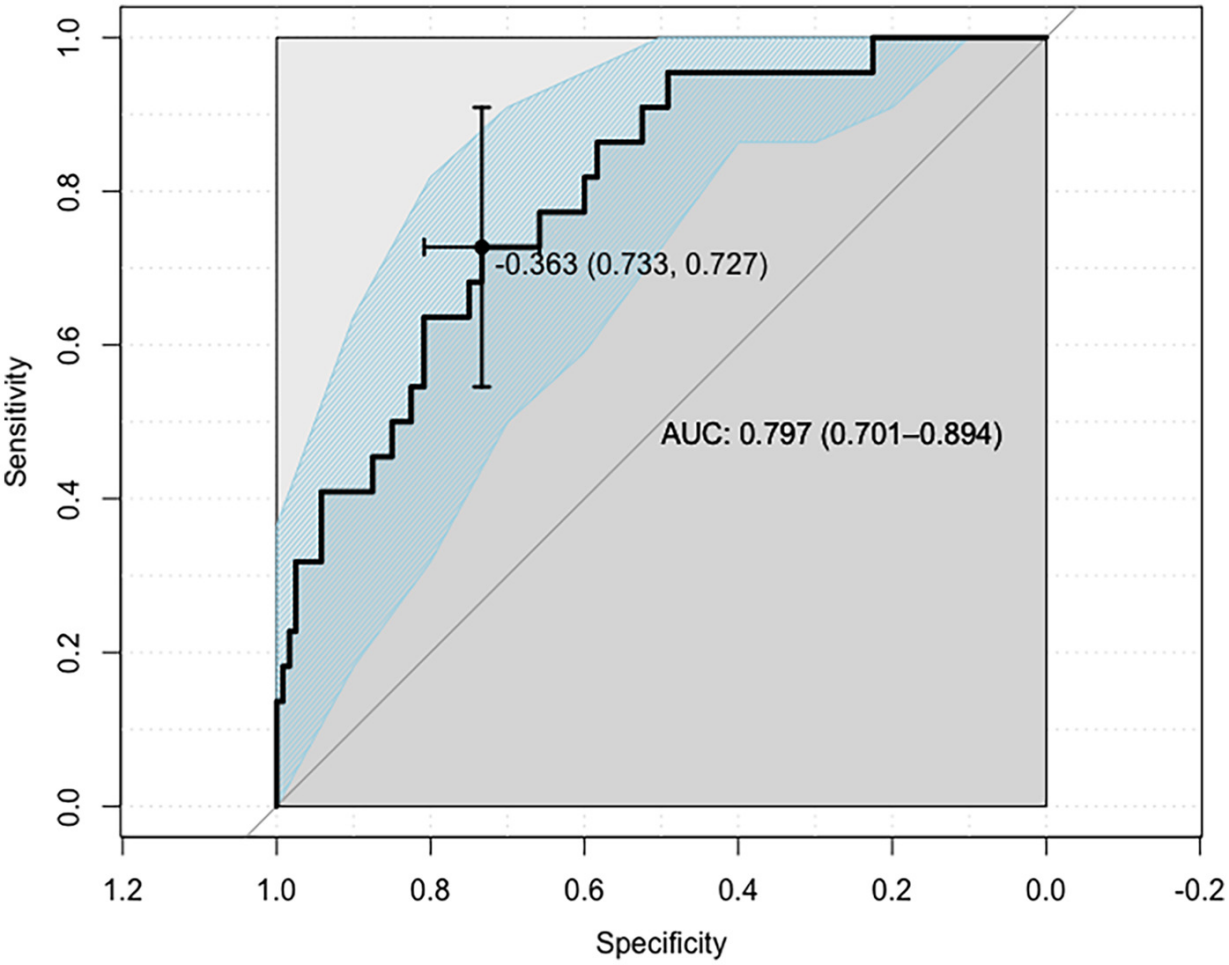
ROC curve defining the cut-off point of the LDF1 variable.

Again, the model refined the classification of patients into one of the aforementioned two groups (OR = 7.3, 95% CI: 2.6–20.4; *p* = 0.0001; Figure 4).

The general classification performance of both models is summarized in Table 4.

**Table 4:** Summary of the general classification performance of model 1 and model 2.

Model	AUC	Sensitivity	Specificity	PPV	NPV	Accuracy of SS ≤ 22 pts	Accuracy of SS ≥ 23 pts	Accuracy (overall)	OR [95% CI]	*p*
Model 1	0.6198 [0.4066–0.833]	0.5455 [0.2727–0.8182]	0.7797 [0.7034–0.8475]	0.1875	0.9485	0.7797	0.5455	0.7597	4.246 [1.199–15.032]	0.027
Model 2	0.7973 [0.7009–0.8938]	0.7273 [0.5455–0.9091]	0.7333 [0.65–0.8083]	0.3333	0.9362	0.7333	0.7273	0.7324	7.333 [2.64–20.373]	0.0001

Abbreviations: AUC, area under the curve; NPV, negative predictive value; PPV, positive predictive value; SS, SYNTAX score.

## 4 Discussion

The findings of our study indicate that the Lp(a) concentration importantly varies depending on the complexity of coronary lesions measured by SS in patients with pCAD. An Lp(a) level of 63.5 mg/dl can be considered the cut-off point for the identification of subjects with severe coronary lesions and SS ≥ 23 points. Moreover, LDA-based modelling allowed the further improvement of such a simple classifier. The LDA model comprised Lp(a), age, sex and LDL-C, displaying refined classification performance in terms of discriminating patients with SS ≥ 23 points from those with SS ≤ 22. Both the Lp(a) threshold and a simple formula based on age, sex, LDL-C and Lp(a) can be used to predict the complexity of coronary artery lesions.

Kaiser et al. found that patients with advanced stable multivessel CAD and Lp(a) > 70 mg/dl undergoing further computed tomography scans experienced the faster progression of low-density plaque volume and fibrofatty plaque volumes than those with low Lp(a). Moreover, multivariate linear regression analysis confirmed the progression of low-attenuation plaque for each 50 mg/dl of Lp(a). High levels of Lp(a) above 70 mg/dl were associated with the accelerated progression of low-attenuation plaque (necrotic core), despite guideline-based preventive therapies (53).

Our cut-off value for the Lp(a) of 63.5 mg/dl is interesting considering the UK Biobank analysis, which demonstrated that in individuals with a history of ASCVD, Lp(a) ≥70 mg/dl was associated with an adjusted hazard ratio for a subsequent ASCVD episode of 1.16 (95% CI, 1.05–1.27). In patients receiving secondary prevention, there was also a statistically significant increase in CAD risk, with an HR of 1.23 (95% CI: 1.10–1.37). An Lp(a) level ≥70 mg/dl (≥150 nmol/L) was also an eligibility criterion for the ongoing Lp(a)HORIZON clinical trial evaluating the effect of Lp(a) lowering with pelacarsen on major cardiovascular events in patients with CVD in a secondary prevention setting (27, 54). The utility of the inclusion of Lp(a) in risk stratification models was emphasized by Welsh et al. They used Cox models to analyze the associations of Lp(a) with composite fatal/non-fatal CVD, fatal CVD, CAD, PAD and aortic stenosis. After adjustment for classical risk factors, a 1 SD increment in log Lp(a) was associated with a hazard ratio for fatal/non-fatal CVD of 1.12 [95% confidence interval (CI), 1.10–1.15]. Similar associations were observed with fatal CVD, CAD, PAD and aortic stenosis. Conversely, adding Lp(a) to a prediction model containing traditional ASCVD risk factors in a primary prevention group augmented the C-index by + 0.0017 (95% CI, 0.0008–0.0026). The authors also noted that the above improvement in the C-index with Lp(a) was approximately four times higher than previously reported for C-reactive protein (55).

Moreover, in 2022, experts proposed considering the Lp(a) level in addition to classical risk factors such as age, gender, BMI, basic lipid parameters and systolic blood pressure in an online calculator estimating the risk of heart attack and stroke (https://www.lpaclinicalguidance.com/) (56).

Similar to Safarian et al., in our analysis, we divided the study group based on the SS threshold indicated in the ESC/EACTS Guidelines on Myocardial Revascularization, which highlight that the cut-off point of SS ≥ 23 has clinical implications for the selection of the revascularization strategy. In patients with multivessel CAD and an SS ≥ 23, the coronary artery bypass grafting technique is preferred, whereas in the SS 0–22 group, PCI is suggested. Additionally, the SS threshold correlates with the prognosis of adverse events (cardiac death, in-hospital mortality, nonfatal MI or target vessel revascularization) following PCI in patients with three-vessel disease (32, 57–59). Although the sample sizes of our study groups were largely unbalanced, a similar epidemiology was observed in the BARI-2D Study, where SS ≥ 23 represented approximately 21% of the study population (60).

Similar to Cappelletti et al., we did not demonstrate significant differences between the identified groups in the incidence of CV risk factors such as HT, obesity, DM, smoking and LDL-C levels (61).

The validity of using SS to assess the prognoses of a relatively young patient population is justified additionally by the analysis of Eickhoff et al., which found that SS is an independent predictor of the risk of death 1–2 years after PCI in most patients approximately 75 years of age (62). In contrast, Lin et al. illustrated a positive correlation between LDL-C and ApoB levels and the severity of coronary lesions assessed by SS (63).

Tzu-Hsiang Lin et al. demonstrated that among CAD patients without prior revascularization, the group of subjects with SS ≥ 23 had a notably higher LDL-C level than the group with low SS. Furthermore, SS ≥ 23 points was associated not only with dyslipidaemia, defined as an elevated LDL-C, TC and TC/HDL-C ratio, but also with a history of statin use before hospitalization and HT. However, the investigators did not assess Lp(a) and its correlation with SS (64). The association between higher levels of Lp(a) and the severity of CAD was demonstrated by both Ooi et al. and Farnier et al. They found that the Lp(a) concentration was associated with both Gensini and CAGE scores and remained important following adjustments for conventional CVD risk factors (65). In addition, in patients hospitalized for myocardial infarction, very high Lp(a) levels were independently associated with a severe CAD burden measured by SS and more frequent multivessel disease (66).

An analysis of patients with ACS and CAD diagnosed at <60 years of age depicted that elevated Lp(a) and LDL-C levels were independent predictors of higher SS scores in this group of patients (67).

Interestingly, the results of another study suggested that the relationship between the Lp(a) and SS score persists only in the LDL-C ≥100 mg/dl group, and Lp(a) >30 mg/dl is an independent predictor of the severity of coronary artery stenoses defined as SS ≥ 23 points only in the LDL-C ≥100 mg/dl group (68). In our study, we did not find that the association between Lp(a) and SS was influenced by LDL-C ≥100 mg/dl and attaining the target LDL-C level of <55 mg/dl.

Although current ESC guidelines for the management of dyslipidaemia recommend that Lp(a) levels should be measured at least once in every adult, in routine practice, the test is performed rarely, even in patients with documented atherosclerosis and very high cardiovascular risk (44, 56, 69).

In a multicenter, cross-sectional epidemiological Lp(a)HERITAGE study to evaluate the incidence of elevated Lp(a) in patients with known ASCVD (post-myocardial infarction, ischemic stroke or PAD), nearly 90% of the obtained Lp(a) results were not known before enrolment (24). Catapano et al. reported that even after a measurement, information about the Lp(a) level is not commonly considered in the management of most patients with ASCVD (70).

The modern healthcare system allows the relatively rapid generation of large and complex datasets to obtain new knowledge about patient care. Identifying effective, useful and generalizable relationships by combining sets of information using advanced analytical methods allows the elucidation of patterns that may be applicable to everyday clinical practice. Pilger et al. pointed out that mathematically combining the analyzed variables (here, laboratory data, including lipid parameters) using LDA can significantly improve the classification of peripheral arterial atherosclerosis (71). The use of Fisher's analysis in designing screening tests based on biochemical and demographic indicators was also highlighted in a retrospective assessment to predict the formation and location of atherosclerotic plaques in carotid arteries (72). Ricciardi et al.'s analysis underscored that the discussion of the practical implementation of data mining techniques and the importance of LDA were useful tools in predicting coronary artery disease and making clinical decisions (73).

We have chosen LDA for its well-known ability to provide a linear combination of predictors that maximally separates groups (classes), which facilitates straightforward interpretation in a clinical context. Additionally, it is computationally efficient and suitable for our sample size, making it preferable to more complex machine-learning methods (e.g., neural networks or ensemble tree-based solutions) that require larger datasets and can be more challenging to interpret. While logistic regression is also commonly used for classification by estimating the probability of an outcome, LDA explicitly aims to identify linear decision boundaries that facilitate more straightforward interpretation, aligning well with classification goals of this study. LDA explicitly identifies linear combinations of variables that maximize separation between groups, while, on the other hand, logistic regression focuses on estimating the probability of an outcome. Although LDA is considered a foundational machine-learning technique for classification, it still does have classical statistical roots. The stepwise procedure we used helps systematically evaluate the contribution of multiple potential predictors, aligning well with the data-driven nature of our investigation. A more detailed discussion of LDA can be found elsewhere (51, 52).

An analysis of 3316 patients enrolled in the Ludwigshafen Risk and Cardiovascular Health (LURIC) study showed that using Lp(a) as one of the laboratory parameters in a machine learning predictive model can reduce the need for invasive diagnostic techniques. A high lipoprotein(a) concentration was positively associated with angiographic CAD (41). Furthermore, the potential of these findings was demonstrated in the extension analysis in Young Finns Study (YFS) (74). The applicability to atherosclerotic vascular disease in a younger population was confirmed (41).

Model 1 distinguished patients with SS ≥ 23 points from patients with SS ≤ 22 points by the Lp(a) cut-off point of 63.5 mg/dl [sensitivity 0.546, specificity 0.780; AUC 0.62 (95% CI: 0.41–0.83); *p* = 0.027]. However, the obtained level of significance implies a limited practical value for the indicated model. Although the AUC is at the 62% level, the 95% confidence interval falls within the value of 50% (95% CI: 41%–83%), which indicates a rather poor overall classification performance. This is confirmed by the low sensitivity (about 55%) and very low PPV of the model, which is about 19%. In contrast, the levels of specificity (78%) and NPV (95%) are satisfactory. The above data suggest that the model based on the designated threshold for Lp(a) is more accurate in identifying patients from the SS ≤ 22-point group (78%), but in fact is very often mistaken in identifying patients from the SS ≥ 23-point group (55%).

An attempt to increase the clinical value of Lp(a) in predicting the severity of coronary artery stenosis was the use of LDA, considering potential confounding variables, followed by ROC analysis. Confounding variables were defined as classic CVD risk factors, including age, sex, BMI, smoking, LDL-C level, statin use, HT, DM (type 1 or 2), positive family history of CAD and stage G3. Model 2, built based on 4 variables—3 continuous predictors [Lp(a), LDL-C, age] and 1 qualitative predictor (gender)—was characterized by comparable accuracy in identifying people from the 2 previously distinguished groups of CAD severity: SS ≤ 22 points and SS ≥ 23 points.

This time, the AUC was 80%, which is higher than in Model 1, based on the Lp(a) variable alone, and the 95% confidence interval did not include the value of 50% (95% CI: 70%–89%), which indicates the relatively good overall classification efficiency of Model 2. Both the sensitivity (73%) and specificity (73%) of the model were considered satisfactory. In this case, the PPV of the model was higher than previously (33%). However, it was still relatively low. The NPV was 94%, considered a satisfactory value. In conclusion, the additional inclusion of parameters other than Lp(a) in the analysis, in this case selected CVD risk factors—age, gender and LDL-C level—allows the creation of a statistical model characterized by comparable accuracy in identifying patients from SS groups ≤22 points and ≥23 points, which may have potential applications in everyday clinical practice. The odds ratio (OR) in Table 4 compares patients’ actual SS classifications with those obtained using models 1 and 2.

The value of the linear discriminant function (LDF1) was calculated according to the above equation. The mean value of LDF1 was higher in the SS ≥ 23 point group than in the SS ≤ 22 point group (Table 1). Thus, higher values for Lp(a), LDL-C and age will result in elevated LDF1 values and a higher probability that the subject will be classified by Model 2 as SS ≥ 23 points. Female gender (“F”) reduces the LDF1 value by 1.2149, which results in a lower probability that a subject will be assigned to the SS ≥ 23 point class. Consequently, the SS ≥ 23-point group will include a higher percentage of subjects with elevated Lp(a) and LDL-C levels, as well as older patients, and more men than women.

Currently available statistical tools include a huge range of multivariate modelling methods. A comprehensive comparison of the obtained models would certainly be beyond the scope of this manuscript and was not its purpose. In our study, we attempted to estimate the predictive ability of a multivariate statistical model based on a suitable and well-known method of linear discriminant function analysis. Further research and modifications using other available methods are reasonable for improving its clinical value.

In summary, we want to underscore the importance of simultaneous measurements of Lp(a) concentrations and lipid profiles in patients undergoing coronary angiography. An Lp(a) level of 63.5 mg/dl can be the cut-off point for the identification of subjects with SS ≥ 23. However, the proposed LDA-based modelling using Lp(a), LDL-C, age and gender may be an applicable tool for the preliminary identification of patients at risk of more complex coronary artery lesions. Based on those findings, we can propose a pragmatic tool to prioritize patients with pCAD qualified for coronary angiography. Implementing a machine-learning approach may simplify the decision-making process in patients with pCAD.

## 5 Limitations

The study population was relatively small, which limits the ability of implementing the results to a wider population. Nevertheless, it should be emphasized that the recruitment was limited significantly by the COVID-19 pandemic ongoing during the collection of the study cohort. The results of the current study should be interpreted with caution, emphasizing the need for additional analyses with larger samples, enabling a more comprehensive assessment of the stability and performance of the model. Although a training set (70% of the data) and a test set (30%) were employed in our LDA modeling, the total number of participants (approximately 150) may not be sufficient to draw definitive conclusions. Consequently, any findings derived from the LDA must be interpreted with particular caution, as the small sample size limits the generalizability and robustness of the results.

Although the SS analysis in our study was performed by two or, in questionable cases, three experienced invasive cardiologists, the possibilities of discrepancies between observers, correlated with the partially subjective nature of the assessment, were previously reported (75).

The strength of our study is the supervised machine learning approach (76).

## Data Availability

The original contributions presented in the study are included in the article/Supplementary Material, further inquiries can be directed to the corresponding author.

## References

[1] Wilmot KA O’Flaherty M Capewell S Ford ES Vaccarino V. Coronary heart disease mortality declines in the United States from 1979 through 2011. Circulation. (2015) 132:997–1002. 10.1161/CIRCULATIONAHA.115.015293 26302759 PMC4828724

[2] Arora S Stouffer GA Kucharska-Newton AM Qamar A Vaduganathan M Pandey A Twenty year trends and sex differences in young adults hospitalized with acute myocardial infarction: the ARIC community surveillance study. Circulation. (2019) 139:1047–56. 10.1161/CIRCULATIONAHA.118.037137 30586725 PMC6380926

[3] Berg K. A new serum type system in man–the Lp system. Acta Pathol Microbiol Scand. (1963) 59:369–82. 10.1111/J.1699-0463.1963.TB01808.X 14064818

[4] Erqou S Kaptoge S Perry PL Di Angelantonio E Thompson A White IR Lipoprotein(a) concentration and the risk of coronary heart disease, stroke, and nonvascular mortality the emerging risk factors collaboration * Europe PMC funders group. JAMA. (2009) 302:412–23. 10.1001/jama.2009.1063.Lipoprotein(a) 19622820 PMC3272390

[5] Nordestgaard BG Chapman MJ Ray K Borén J Andreotti F Watts GF Lipoprotein(a) as a cardiovascular risk factor: current status. Eur Heart J. (2010) 31:2844–53. 10.1093/EURHEARTJ/EHQ386 20965889 PMC3295201

[6] Bhatia HS Becker RC Leibundgut G Patel M Lacaze P Tonkin A Lipoprotein(a), platelet function and cardiovascular disease. Nat Rev Cardiol. (2024) 21(5):299–311. 10.1038/s41569-023-00947-2 37938756 PMC11216952

[7] Gu J-X Huang J Li S-S Zhou L-H Yang M Li Y Elevated lipoprotein(a) and genetic polymorphisms in the LPA gene may predict cardiovascular events. Sci Rep. (2022) 12:3588. 10.1038/s41598-022-07596-4 35246583 PMC8897417

[8] Sosnowska B Stepinska J Mitkowski P Bielecka-Dabrowa A Bobrowska B Budzianowski J Recommendations of the experts of the Polish Cardiac Society (PCS) and the Polish Lipid Association (PoLA) on the diagnosis and management of elevated lipoprotein(a) levels. Arch Med Sci. (2024) 20:8–27. 10.5114/aoms/183522 38414479 PMC10895977

[9] Nielsen LB Stender S Kjeldsen K Nordestgaard BG. Specific accumulation of lipoprotein(a) in balloon-injured rabbit aorta *in vivo*. Circ Res. (1996) 78:615–26. 10.1161/01.RES.78.4.615 8635219

[10] Tsimikas S Fazio S Ferdinand KC Ginsberg HN Koschinsky ML Marcovina SM NHLBI working group recommendations to reduce lipoprotein(a)-mediated risk of cardiovascular disease and aortic stenosis. J Am Coll Cardiol. (2018) 71:177–92. 10.1016/J.JACC.2017.11.014 29325642 PMC5868960

[11] Group SSSS. Randomised trial of cholesterol lowering in 4444 patients with coronary heart disease: the scandinavian simvastatin survival study (4S). Lancet. (1994) 344:1383–9. 10.1016/S0140-6736(94)90566-5 7968073

[12] Albers JJ Slee A O’Brien KD Robinson JG Kashyap ML Kwiterovich PO Relationship of apolipoproteins A-1 and B, and lipoprotein(a) to cardiovascular outcomes: the AIM-HIGH trial (atherothrombosis intervention in metabolic syndrome with low HDL/high triglyceride and impact on global health outcomes). J Am Coll Cardiol. (2013) 62:1575–9. 10.1016/J.JACC.2013.06.051 23973688 PMC3800510

[13] Khera AV Everett BM Caulfield MP Hantash FM Wohlgemuth J Ridker PM Lipoprotein(a) concentrations, rosuvastatin therapy, and residual vascular risk: an analysis from the JUPITER trial (justification for the use of statins in prevention: an intervention trial evaluating rosuvastatin). Circulation. (2014) 129:635–42. 10.1161/CIRCULATIONAHA.113.004406 24243886 PMC3946056

[14] LIPID Study Group (Long-term Intervention with Pravastatin in Ischaemic Disease). Long-term effectiveness and safety of pravastatin in 9014 patients with coronary heart disease and average cholesterol concentrations: the LIPID trial follow-up. Lancet. (2002) 359(9315):1379–87. 10.1016/S0140-6736(02)08351-4. Erratum in: *Lancet* (2002) 360(9343):1430. 11978335

[15] Sabatine MS Giugliano RP Keech AC Honarpour N Wiviott SD Murphy SA Evolocumab and clinical outcomes in patients with cardiovascular disease. N Engl J Med. (2017) 376:1713–22. 10.1056/NEJMoa1615664 28304224

[16] Pederiva C Capra ME Biasucci G Banderali G Fabrizi E Gazzotti M Lipoprotein(a) and family history for cardiovascular disease in paediatric patients: a new frontier in cardiovascular risk stratification. Data from the LIPIGEN paediatric group. Atherosclerosis. (2022) 349:233–9. 10.1016/j.atherosclerosis.2022.04.021 35562202

[17] Thomas PE Vedel-Krogh S Kamstrup PR Nordestgaard BG. Lipoprotein(a) is linked to atherothrombosis and aortic valve stenosis independent of C-reactive protein. Eur Heart J. (2023) 44:1449–60. 10.1093/eurheartj/ehad055 36805188

[18] Kumar P Swarnkar P Misra S Nath M. Lipoprotein (a) level as a risk factor for stroke and its subtype: a systematic review and meta-analysis. Sci Rep. (2021) 11:15660. 10.1038/s41598-021-95141-0 34341405 PMC8329213

[19] Dhindsa DS Sandesara PB Shapiro MD Wong ND. The evolving understanding and approach to residual cardiovascular risk management. Front Cardiovasc Med. (2020) 7:88. 10.3389/fcvm.2020.00088 32478100 PMC7237700

[20] Ober C Nord AS Thompson EE Pan L Tan Z Cusanovich D Genome-wide association study of plasma lipoprotein(a) levels identifies multiple genes on chromosome 6q. J Lipid Res. (2009) 50:798–806. 10.1194/JLR.M800515-JLR200 19124843 PMC2666166

[21] Zabaneh D Kumari M Sandhu M Wareham N Wainwright N Papamarkou T Meta analysis of candidate gene variants outside the LPA locus with Lp(a) plasma levels in 14,500 participants of six white European cohorts. Atherosclerosis. (2011) 217:447–51. 10.1016/J.ATHEROSCLEROSIS.2011.04.015 21592478 PMC3972487

[22] Schmidt K Noureen A Kronenberg F Utermann G. Structure, function, and genetics of lipoprotein (a). J Lipid Res. (2016) 57:1339–59. 10.1194/JLR.R067314 27074913 PMC4959873

[23] Lacaze P Bakshi A Riaz M Polekhina G Owen A Bhatia HS Aspirin for primary prevention of cardiovascular events in relation to lipoprotein(a) genotypes. J Am Coll Cardiol. (2022) 80:1287–98. 10.1016/J.JACC.2022.07.027 36175048 PMC10025998

[24] Nissen SE Wolski K Cho L Nicholls SJ Kastelein J Leitersdorf E Lipoprotein(a) levels in a global population with established atherosclerotic cardiovascular disease. Open Hear. (2022) 9:e002060. 10.1136/openhrt-2022-002060 PMC9577925 36252994

[25] Finneran P Pampana A Khetarpal SA Trinder M Patel AP Paruchuri K Lipoprotein(a) and coronary artery disease risk without a family history of heart disease. J Am Heart Assoc. (2021) 10:e017470. 10.1161/JAHA.120.017470 33631942 PMC8174293

[26] Korneva V Kuznetsova T Julius U. Analysis of lipid metabolism and its impact on the risk of ischemic heart disease in patients with definite familial hypercholesterolemia. Atheroscler Suppl. (2017) 30:56–62. 10.1016/j.atherosclerosissup.2017.05.008 29096862

[27] Patel AP Wang M Pirruccello JP Ellinor PT Ng K Kathiresan S Arteriosclerosis, thrombosis, and vascular biology Lp(a) [lipoprotein(a)] concentrations and incident atherosclerotic cardiovascular disease new insights from a large national biobank. Arter Thromb Vasc Biol. (2021) 41:465–74. 10.1161/ATVBAHA.120.315291 PMC7769893 33115266

[28] Rallidis LS Pavlakis G Foscolou A Kotakos C Katsimardos A Drosatos A High levels of lipoprotein (a) and premature acute coronary syndrome. Atherosclerosis. (2018) 269:29–34. 10.1016/j.atherosclerosis.2017.12.011 29258004

[29] Knuuti J Wijns W Saraste A Capodanno D Barbato E Funck-Brentano C ESC guidelines for the diagnosis and management of chronic coronary syndromes: the task force for the diagnosis and management of chronic coronary syndromes of the European Society of Cardiology (ESC). Eur Heart J. (2019) 2020(41):407–77. 10.1093/eurheartj/ehz425 31504439

[30] Zaman T Agarwal S Anabtawi AG Patel NS Ellis SG Tuzcu EM Angiographic lesion severity and subsequent myocardial infarction. Am J Cardiol. (2012) 110:167–72. 10.1016/j.amjcard.2012.03.008 22497675

[31] Ashfaq F Goel PK Moorthy N Sethi R Idrees Khan M Idris MZ. Lipoprotein(a) and SYNTAX score association with severity of coronary artery atherosclerosis in north India. Sultan Qaboos Univ Med J. (2012) 12:465–72. 10.12816/0003172 23275843 PMC3523996

[32] Sousa-Uva M Neumann F-J Ahlsson A Alfonso F Banning AP Benedetto U 2018 ESC/EACTS guidelines on myocardial revascularization. Eur J Cardio Thoracic Surg. (2019) 55:4–90. 10.1093/ejcts/ezy289 30165632

[33] Chen J Zhang Y Liu J Chen M-H Guo Y-L Zhu C-G Role of lipoprotein(a) in predicting the severity of new on-set coronary artery disease in type 2 diabetics: a gensini score evaluation. Diabetes Vasc Dis Res. (2015) 12:258–64. 10.1177/1479164115579004 25861813

[34] Wykrzykowska JJ Garg S Girasis C de Vries T Morel M-A van Es G-A Value of the SYNTAX score for risk assessment in the all-comers population of the randomized multicenter LEADERS (limus eluted from a durable versus ERodable stent coating) trial. J Am Coll Cardiol. (2010) 56:272–7. 10.1016/j.jacc.2010.03.044 20633818

[35] Garg S Serruys PW Silber S Wykrzykowska J van Geuns RJ Richardt G The prognostic utility of the SYNTAX score on 1-year outcomes after revascularization with zotarolimus- and everolimus-eluting stents: a substudy of the RESOLUTE all comers trial. JACC Cardiovasc Interv. (2011) 4:432–41. 10.1016/j.jcin.2011.01.008 21511223

[36] Zhao M Stampf S Valina C Kienzle R-P Ferenc M Gick M Role of euroSCORE II in predicting long-term outcome after percutaneous catheter intervention for coronary triple vessel disease or left main stenosis. Int J Cardiol. (2013) 168:3273–9. 10.1016/j.ijcard.2013.04.136 23664045

[37] Cavalcante R Sotomi Y Mancone M Whan Lee C Ahn J-M Onuma Y Impact of the SYNTAX scores I and II in patients with diabetes and multivessel coronary disease: a pooled analysis of patient level data from the SYNTAX, PRECOMBAT, and BEST trials. Eur Heart J. (2017) 38:1969–77. 10.1093/eurheartj/ehx138 28431047

[38] Serruys PW Morice M-C Kappetein AP Colombo A Holmes DR Mack MJ Percutaneous coronary intervention versus coronary-artery bypass grafting for severe coronary artery disease. N Engl J Med. (2009) 360:961–72. 10.1056/NEJMoa0804626 19228612

[39] Alizadehsani R Abdar M Roshanzamir M Khosravi A Kebria PM Khozeimeh F Machine learning-based coronary artery disease diagnosis: a comprehensive review. Comput Biol Med. (2019) 111:103346. 10.1016/J.COMPBIOMED.2019.103346 31288140

[40] Sayadi M Varadarajan V Sadoughi F Chopannejad S Langarizadeh M. A machine learning model for detection of coronary artery disease using noninvasive clinical parameters. Life. (2022) 12:1933. 10.3390/LIFE12111933 36431068 PMC9698583

[41] Koloi A Loukas VS Hourican C Sakellarios AI Quax R Mishra PP Predicting early-stage coronary artery disease using machine learning and routine clinical biomarkers improved by augmented virtual data. Eur Hear J Digit Heal. (2024) 5:542. 10.1093/EHJDH/ZTAE049 PMC11417487 39318697

[42] Arnett DK Blumenthal RS Albert MA Buroker AB Goldberger ZD Hahn EJ 2019 ACC/AHA guideline on the primary prevention of cardiovascular disease: executive summary: a report of the American College of Cardiology/American Heart Association task force on clinical practice guidelines. Circulation. (2019) 140:e563–95. 10.1161/CIR.0000000000000677 30879339 PMC8351755

[43] Friedewald WT Levy RI Fredrickson DS. Estimation of the concentration of low-density lipoprotein cholesterol in plasma, without use of the preparative ultracentrifuge. Clin Chem. (1972) 18:499–502. 10.1093/clinchem/18.6.499 4337382

[44] Mach F Baigent C Catapano AL Koskinas KC Casula M Badimon L 2019 ESC/EAS guidelines for the management of dyslipidaemias: lipid modification to reduce cardiovascular risk: the task force for the management of dyslipidaemias of the European Society of Cardiology (ESC) and European atherosclerosis society (EAS). Eur Heart J. (2019) 41:111–88. 10.1093/eurheartj/ehz455 31504418

[45] Rerkasem A Orrapin S Howard DP Rerkasem K. Carotid endarterectomy for symptomatic carotid stenosis. Cochrane Database Syst Rev. (2020) 9(9):CD001081. 10.1002/14651858.CD001081.pub4 32918282 PMC8536099

[46] Goldstein LB. Screening for asymptomatic carotid artery stenosis: lack of clinical benefit, potential for harm. JAMA. (2021) 325:443–4. 10.1001/JAMA.2020.26440 33528518

[47] Krist AH Davidson KW Mangione CM Barry MJ Cabana M Caughey AB Screening for asymptomatic carotid artery stenosis: US preventive services task force recommendation statement. JAMA. (2021) 325:476–81. 10.1001/JAMA.2020.26988 33528542

[48] Visseren FLJ Mach F Smulders YM Carballo D Koskinas KC Bäck M ESC guidelines on cardiovascular disease prevention in clinical practice: developed by the task force for cardiovascular disease prevention in clinical practice with representatives of the European Society of Cardiology and 12 medical societies with. Eur Heart J. (2021) 2021(42):3227–337. 10.1093/eurheartj/ehab484 34458905

[49] WHO. Noncommunicable diseases: risk factors (n.d). Available at: https://www.who.int/data/gho/data/themes/topics/topic-details/GHO/ncd-risk-factors (Accessed June 11, 2023).

[50] R Core Team. R: A Language and Environment for Statistical Computing. Vienna: R Foundation for Statistical Computing (2021).

[51] Fisher RA. The use of multiple measurements in taxonomic problems. Ann Eugen. (1936) 7:179–88. 10.1111/J.1469-1809.1936.TB02137.X

[52] Stanisz A. Przystępny Kurs Statystyki z Zastosowaniem STATISTICA PL na Przykładach z Medycyny. Tom 3: Analizy Wielowymiarowe. Kraków: StatSoft Polska (2007).

[53] Kaiser Y Daghem M Tzolos E Meah MN Doris MK Moss AJ Association of lipoprotein(a) with atherosclerotic plaque progression. J Am Coll Cardiol. (2022) 79:223–33. 10.1016/j.jacc.2021.10.044 35057907 PMC8784819

[54] Malick WA Goonewardena SN Koenig W Rosenson RS. Clinical trial design for lipoprotein(a)-lowering therapies: JACC focus seminar 2/3. J Am Coll Cardiol. (2023) 81:1633–45. 10.1016/j.jacc.2023.02.033 37076218

[55] Welsh P Welsh C Celis-Morales CA Brown R Ho FK Ferguson LD Lipoprotein(a) and cardiovascular disease: prediction, attributable risk fraction, and estimating benefits from novel interventions. Eur J Prev Cardiol. (2021) 28:1991–2000. 10.1093/eurjpc/zwaa063 33624048

[56] Kronenberg F Mora S Stroes ESG Ference BA Arsenault BJ Berglund L Lipoprotein(a) in atherosclerotic cardiovascular disease and aortic stenosis: a European atherosclerosis society consensus statement. Eur Heart J. (2022) 43:3925–46. 10.1093/eurheartj/ehac361 36036785 PMC9639807

[57] Xu N Tang XF Yao Y Jia DS Liu Y Zhao XY Lipoprotein(a) levels are associated with coronary severity but not with outcomes in Chinese patients underwent percutaneous coronary intervention. Nutr Metab Cardiovasc Dis. (2020) 30(2):265–73. 10.1016/j.numecd.2019.09.020 31740238

[58] Sianos G Morel M-A Kappetein AP Morice M-C Colombo A Dawkins K The SYNTAX score: an angiographic tool grading the complexity of coronary artery disease. EuroIntervention. (2005) 1:219–27. 19758907

[59] Safarian H Alidoosti M Shafiee A Salarifar M Poorhosseini H Nematipour E. The SYNTAX score can predict major adverse cardiac events following percutaneous coronary intervention. Hear Views. (2014) 15:99–105. 10.4103/1995-705X.151081 PMC4348991 25774251

[60] Ikeno F Brooks M Nakagawa K Kim M-K Kaneda H Mitsutake Y SYNTAX score and long-term outcomes. J Am Coll Cardiol. (2017) 69:395–403. 10.1016/j.jacc.2016.10.067 28126156

[61] Cappelletti A Astore D Godino C Bellini B Magni V Mazzavillani M Relationship between syntax score and prognostic localization of coronary artery lesions with conventional risk factors, plasma profile markers, and carotid atherosclerosis (CAPP study 2). Int J Cardiol. (2018) 257:306–11. 10.1016/j.ijcard.2017.12.012 29506713

[62] Age-dependent impact of the SYNTAX-score on longer-term mortality after percutaneous coronary intervention in an all-comer population. J Geriatr Cardiol. (2018) 15:559–66. 10.11909/j.issn.1671-5411.2018.09.009 30344539 PMC6188979

[63] Lin T Wang L Guo J Liu P Chen L Wei M Association between Serum LDL-C and ApoB and SYNTAX score in patients with stable coronary artery disease. Angiology. (2018) 69:724–9. 10.1177/0003319717748771 29310455

[64] Lin T-H Lee W-L Lee W-J Sheu WH-H Liao Y-C Liang K-W. Dyslipidemia, not inflammatory markers or adipokines, contributes significantly to a higher SYNTAX score in stable coronary artery disease (from the Taichung CAD study). Acta Cardiol Sin. (2021) 37(3):232–8. 10.6515/ACS.202105_37(3).20201116B 33976506 PMC8107712

[65] Ooi EMM Ellis KL Barrett PHR Watts GF Hung J Beilby JP Lipoprotein(a) and apolipoprotein(a) isoform size: associations with angiographic extent and severity of coronary artery disease, and carotid artery plaque. Atherosclerosis. (2018) 275:232–8. 10.1016/j.atherosclerosis.2018.06.863 29960898

[66] Farnier M Chagué F Maza M Bichat F Masson D Cottin Y High lipoprotein(a) levels predict severity of coronary artery disease in patients hospitalized for acute myocardial infarction. Data from the French RICO survey. J Clin Lipidol. (2022) 16:685–93. 10.1016/j.jacl.2022.07.006 35995726

[67] Chieng D Pang J Ellis KL Hillis GS Watts GF Schultz CJ. Elevated lipoprotein(a) and low-density lipoprotein cholesterol as predictors of the severity and complexity of angiographic lesions in patients with premature coronary artery disease. J Clin Lipidol. (2018) 12:1019–26. 10.1016/j.jacl.2018.03.090 29703625

[68] Xu W Guan H Gao D Wang Z Ba Y Yang H The association of syntax score with levels of lipoprotein(a) and inflammatory biomarkers in patients with stable coronary artery disease and different low-density lipoprotein cholesterol levels. Diabetes Metab Syndr Obes. (2020) 13:4297–310. 10.2147/DMSO.S279814 33209043 PMC7669512

[69] Kronenberg F Mora S Stroes ESG Ference BA Arsenault BJ Berglund L Frequent questions and responses on the 2022 lipoprotein(a) consensus statement of the European atherosclerosis society. Atherosclerosis. (2023) 374:107–20. 10.1016/j.atherosclerosis.2023.04.012 37188555

[70] Catapano AL Daccord M Damato E Humphries SE Neely RDG Nordestgaard BG How should public health recommendations address lp(a) measurement, a causative risk factor for cardiovascular disease (CVD)? Atherosclerosis. (2022) 349:136–43. 10.1016/J.ATHEROSCLEROSIS.2022.02.013 35292153

[71] Pilger E Pristautz H Pfeiffer KP Kostner G. Risk factors for peripheral atherosclerosis. Retrospective evaluation by stepwise discriminant analysis. Arteriosclerosis. (1983) 3:57–63. 10.1161/01.ATV.3.1.57 6401992

[72] Hu J Su F Ren X Cao L Zhou Y Fu Y Prediction of carotid plaque by blood biochemical indices and related factors based on fisher discriminant analysis. BMC Cardiovasc Disord. (2022) 22(1):371. 10.1186/S12872-022-02806-3 35965318 PMC9377085

[73] Ricciardi C Valente AS Edmund K Cantoni V Green R Fiorillo A Linear discriminant analysis and principal component analysis to predict coronary artery disease. Health Informatics J. (2020) 26:2181–92. 10.1177/1460458219899210 31969043

[74] Raitakari OT Juonala M Rönnemaa T Keltikangas-Järvinen L Räsänen L Pietikäinen M Cohort profile: the cardiovascular risk in young Finns study. Int J Epidemiol. (2008) 37:1220–6. 10.1093/IJE/DYM225 18263651

[75] Zhang YJ Iqbal J Campos CM Klaveren D V Bourantas C V Dawkins KD Prognostic value of site SYNTAX score and rationale for combining anatomic and clinical factors in decision making: insights from the SYNTAX trial. J Am Coll Cardiol. (2014) 64:423–32. 10.1016/J.JACC.2014.05.022 25082573

[76] Han S Sohn TJ Ng BP Park C. Predicting unplanned readmission due to cardiovascular disease in hospitalized patients with cancer: a machine learning approach. Sci Rep. (2023) 13:13491. 10.1038/s41598-023-40552-4 37596346 PMC10439193

