# Supplementary material for: Lipoproteins predicting coronary lesion complexity in premature coronary artery disease: a supervised machine learning approach
Source: Front Cardiovasc Med. 2025 Apr 24;12:1470500. doi: 10.3389/fcvm.2025.1470500 (PMC12058860; doi:10.3389/fcvm.2025.1470500)
Supplement: Supplementary file 1 [file Table1.pdf]

Supplementary Material

Table S1. Clinical characteristics of the study group (n = 162).

Parameter	n	%
Hypercholesterolaemia	162	100.0
Hypertension	123	75.9
Overweight (BMI ≥ 25 < 30)	65	40.1
Obesity (BMI ≥30)	68	42.0
Smoking	114	70.4
DM2	48	29.6
DM1	2	1.2
Positive family history of pCAD	61	37.7
Prior ACS	75	46.3
Prior PCI and/or CABG	94	58.0
Other ASCVD	25	15.4
Prior stroke/TIA	6	3.7
Carotid atherosclerosis	5	3.1
PAD	15	9.3
CKD stage G3	16	9.9
Asthma	10	6.2
CKD	6	3.7
Prior LLT	124	76.5
LDL-C <55 mg/dl	14	8.6
LDL-C ≥55 mg/dl	147	90.7

Abbreviations: ACS - acute coronary syndrome; ASCVD - atherosclerotic cardiovascular disease; BMI - body mass index; CKD - chronic kidney disease; COPD - chronic obstructive pulmonary disease; DM1 - type 1 diabetes mellitus; DM2 - type 2 diabetes mellitus; LDL-C - low-density lipoprotein-cholesterol; LLT - lipid-lowering treatment; PAD - peripheral arterial disease; pCAD - premature coronary artery disease; PCI - percutaneous coronary intervention; TIA - transient ischemic attack
