# Supplementary material for: Lipoproteins predicting coronary lesion complexity in premature coronary artery disease: a supervised machine learning approach
Source: Front Cardiovasc Med. 2025 Apr 24;12:1470500. doi: 10.3389/fcvm.2025.1470500 (PMC12058860; doi:10.3389/fcvm.2025.1470500)
Supplement: Supplementary file 2 [file Table2.pdf]

Table S2. Biochemical characteristics of the study group (n = 162).

Parameter	Median	IQR
TC [mg/dL]	167.0	137.0–200.0
LDL-C [mg/dL]	89.0	71.0–126.0
HDL-C [mg/dL]	46.0	38.0–54.0
non-HDL-C [mg/dL]	117.0	94.0–152.0
TG [mg/dL]	116.0	85.0–163.0
Lp(a) [mg/dL]	25.0	8.0–69.5
Creatinine [mg/dL]	0.8	0.7–1.0
GFR [mL/min/1.73 m^2]	90.0	79.0–90.0
ALT [IU/L]	26.0	20.0–34.0
CRP [mg/L]	2.0	0.8–3.4
Uric acid [mg/dL]	6.0	4.9–6.6
Glucose [mg/dL]	100.0	92.0–116.3

Abbreviations: ALT - alanine transaminase; CRP - C-reactive protein; GFR - glomerular filtration rate; HDL-C - high-density lipoprotein-cholesterol; IQR - interquartile range; LDL-C - low-density lipoprotein-cholesterol; Lp(a) - lipoprotein(a); non-HDL-C - non-high-density lipoprotein-cholesterol; TC - total cholesterol; TG - triglycerides.
